# Characteristics of sarcopenia subjects in arterial pulse spectrum analysis

**DOI:** 10.3389/fpubh.2022.969424

**Published:** 2022-09-06

**Authors:** Te Ou Young, Li-Wei Wu, Hsin Hsiu, Tao-Chun Peng, Wei-Liang Chen

**Affiliations:** ^1^Division of Family Medicine, Department of Family and Community Medicine, Tri-Service General Hospital, Taipei, Taiwan; ^2^School of Medicine, National Defense Medical Center, Taipei, Taiwan; ^3^Health Management Center, Department of Family and Community Medicine, Tri-Service General Hospital, National Defense Medical Center, Taipei, Taiwan; ^4^Graduate Institute of Biomedical Engineering, National Taiwan University of Science and Technology, Taipei, Taiwan

**Keywords:** sarcopenia, photoplethysmography (PPG), harmonic index, vascular elasticity index (VEI), geriatrics

## Abstract

**Aims:**

Sarcopenia is significantly associated with the number of cardiovascular and metabolic diseases, however, the underlying pathophysiological processes are largely unknown. This study performed harmonic index of finger photoplethysmography (PPG) waveforms with the aims of distinguishing different arterial pulse waveform signals between sarcopenia, presarcopenia, dynapenia, and healthy subjects.

**Methods:**

Sixty-eight subjects were enrolled and obtained 1-min PPG signals, then were assigned to four age-matched groups: control, dynapenia, presarcopenia, and sarcopenia which definition according to Asian Working Group for Sarcopenia (AWGS): 2019 Consensus Update on Sarcopenia Diagnosis and Treatment. Harmonics 1–10 of the PPG waveform were obtained and calculated each of the amplitude proportions (C_*n*_), standard deviations (SD_*n*_), coefficients of variations (CV_*n*_), and vascular elasticity index (VEI) for to evaluating the blood-pressure harmonic variability.

**Results:**

The prevalence of sarcopenia in women gender (8 out of 9, 88.9%, *p* = 0.046) and osteoporosis in dynapenia (7 out of 16, 43.8%, *p* = 0.005) were significant higher. Among the four groups, compared with control, dynapenia, and presarcopenia, sarcopenia had largest SD_*n*_-values for harmonics 1, 2, 3, and 5 (ratio 1, 2, 3, 5 = 0.354, 0.209, 0.137, 0.074); whereas sarcopenia had largest coefficients of variations (CV_*n*_) values for harmonics 1, 2, 3 and 10 (ratio 1, 2, 3, 10 = 0.263, 0.310, 0.402, 0.791). Besides, the Δ odds ratio of ratio 3, 4,and 6 tertile values were significantly increased in sarcopenia and possible sarcopenia group compared with control group. Subjects with sarcopenia had significantly higher VEI in mean, SD, and CV of the PPG waveform (mean = 2.332, SD = 1.479, CV = 0.634, *p* = 0.007) among the groups and the results of binary logistic regression analysis in the tertiles met statistical significance between the sarcopenia and non-sarcopenia groups whether adjusted or unadjusted (adjusted odds ratio 6.956, *p* = 0.030, unadjusted odds ratio 3.937, *p* = 0.039).

**Conclusions:**

The elasticity of vessels among sarcopenia groups in lower-frequency components of harmonic ratio in which we defined as VEI showed a significantly highest VEI mean, SD, and CV in sarcopenia indicates the poorer elasticity of the arteries. The present findings showed finger PPG waveform measurements may be useful for early detection of vascular diseases with patients with sarcopenia in a non-invasive and easy-to-perform technique which may expand the clinical applicability in the future.

## Introduction

In the era of population aging, the number of elderly people is increasing rapidly, which brings a heavy burden to medical care systems and the social economy (1). The aging process leads to the loss of skeletal muscle mass, strength, and physical performance (2, 3). Patients screened for sarcopenia can be classified as healthy or having presarcopenia, dynapenia, or sarcopenia, based on muscle mass, grip strength, and walking speed (4). Sarcopenia is usually accompanied by an increased risk of falls, fractures, hospitalization, weakness, decreased quality of life, and even death (5, 6). It has been suggested that one of the major risk factors for cardiovascular disease is aging (7, 8). Many studies have also shown that lower body muscle mass increases the risk of cardiovascular disease mortality (9–11).

Photoplethysmography (PPG) is an optical technique for detecting microvascular blood volume changes in peripheral tissues, arterial pressure, and compliance. The changes in blood vessel volume caused by blood pressure and pulse changes can be translated to PPG signals. Therefore, the PPG signal has been considered to produce many indicators that can be used for clinical non-invasive detection of the cardiovascular condition. Awad et al. found that the pulse width change of the PPG waveform is related to the change of the peripheral vascular resistance (12), and the dual PPG signal calculating the pulse transit time could be used to detect blood pressure or the pulse wave velocity (13, 14). Frequency-domain analysis in lower-frequency bands of the PPG signal was found to be an effective application for studying autonomic nervous control of the peripheral circulation (15) and characteristics of the hypovolemic response (16).

Due to the quasiperiodicity of the heartbeat, harmonic analysis has become an ideal method for cardiovascular signal analysis, including blood pressure and PPG waveforms, in many frequency domain analysis methods (17, 18). In previous studies, the use of PPG measurements and harmonic component analysis showed that adequate contact pressure can be used to reconstruct and improve the BP waveform signal with the PPG waveform (19), and changing the wave transmission and arterial stiffness by mild cold stimulation on the skin surface could significantly change several harmonic component indices of PPG waveforms (20).

This study performed harmonic index analysis of finger PPG waveforms with the aim of distinguishing different arterial pulse waveform signals between healthy subjects and individuals with sarcopenia, presarcopenia, and dynapenia. Measurements and analysis of the dynapenia and presarcopenia groups were also evaluated for potential vascular hydrodynamic differences that could be detected by PPG in early stage of cardiovascular disease. The information obtained from finger PPG waveform measurements might be useful for the early detection of vascular diseases in patients with sarcopenia, and PPG is a non-invasive and easy-to-perform technique which may expand its clinical applicability in the future.

## Materials and methods

The study was approved by the institutional review board of Tri-Service General Hospital (TSGHIRB 2-108-05-161), which enrolled 68 volunteers; details of the subjects are listed in **Table 2**. Between August 2020 and November 2020, adults aged at least 60 years (71.47 ± 5.222) who came to the Health Management Center of Tri-Service General Hospital for a geriatric health check-up were eligible. The exclusion criteria were as follows: (1) severe illnesses such as heart failure, liver cirrhosis, end-stage renal disease, cerebral vascular diseases, or cancer, (2) severe mental illnesses, such as severe dementia or psychiatric disease, (3) severe endocrine diseases, such as hyper or hypothyroidism, (4) skin lesions that interfere with PPG signal recording, and (5) medications that may interfere with endothelial functioning. All the participants were enrolled voluntarily and were informed and signed the consent form. According to the criteria listed in Table 1, the subjects were assigned to four age-matched groups: control, dynapenia, presarcopenia, and sarcopenia; the labels were defined according to the Asian Working Group for Sarcopenia (AWGS): 2019 Consensus Update on Sarcopenia Diagnosis and Treatment.

**Table 1:** Criteria of control, dynapenia, presarcopenia, and sarcopenia.

Control	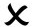	Low handgrip strength (M: <28 kg, F: <18 kg) or Slow 6-m walk: <1.0 m/s
	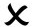	Low appendicular skeletal muscle mass measured by Bioelectrical impedance analysis (M: <7.0 kg/m^2^, F: <5.7 kg/m^2^)
Dynapenia	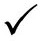	Low handgrip strength (M: <28 kg, F: <18 kg) or Slow 6-m walk: <1.0 m/s
	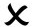	Low appendicular skeletal muscle mass measured by Bioelectrical impedance analysis (M: <7.0 kg/m^2^, F: <5.7 kg/m^2^)
Presarcopenia	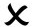	Low handgrip strength (M: <28 kg, F: <18 kg) or Slow 6-m walk: <1.0 m/s
	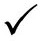	Low appendicular skeletal muscle mass measured by Bioelectrical impedance analysis (M: <7.0 kg/m^2^, F: <5.7 kg/m^2^)
Sarcopenia	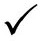	Low handgrip strength (M: <28 kg, F: <18 kg) or Slow 6-m walk: <1.0 m/s
	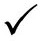	Low appendicular skeletal muscle mass measured by Bioelectrical impedance analysis (M: <7.0 kg/m^2^, F: <5.7 kg/m^2^)

According to Asian Working Group for Sarcopenia: 2019 Consensus Update on Sarcopenia Diagnosis and Treatment.

The participants were all native Taiwanese citizens, wore light clothing, were in the supine position and were allowed to stabilize for at least 10 min before starting the recording. Informed consent was obtained from each participant after receiving an explanation of the study. The subjects' psychological states and moods were assessed in stable condition to prevent interference. The room temperature was between 24 and 26°C during the measurement period. All subjects were required to stop medication (except essential medication such as antihypertensive agents, etc.) for 3 days before the experiment and fasted for at least 1 h before each experiment. All subjects did not drink coffee or alcoholic beverages for at least 1 day before the experiment (19, 20).

### Experimental procedure

The details of the measurements were described in our previous study (19, 20). The ECG signals were measured non-invasively through surface electrodes with a preamplifier (Lead II, RA-LL; 6600 series, Gould, USA). The 940 nm wavelength infrared LED (QED233, Fairchild Optoelectronic) emitted from the PPG device penetrated the middle finger tissue and was collected by a photodiode (L-SB1R9PD1D1, Para, Japan). The applied contact pressure was precalibrated to approximately 60 mmHg to eliminate wearing discomfort and stabilize the PPG measurement. Signals were collected via a current-to-voltage converter circuit and then converted from analog to digital signals at a sampling frequency of 1,024 Hz by a converter card (PCI-9111DG, Adlink Technology, Taiwan). For each measurement, 1-min data series were recorded after measuring the subjects' basic physiological parameters, including systolic blood pressure (SBP), diastolic blood pressure (DBP), and HR, using a sphygmomanometer (MediGuard 150i, Rossmax).

The skin surface temperature was monitored by a thermistor attached around the wrist. By turning the resistance of the thermistor into voltages, signals were sent to an analog-to-digital converter card. The temperature was monitored properly during the measurement, and the variations in temperature were within 1.0°C. The average monitored environmental temperatures were 28.7 ± 1.8°C, 29.1 ± 1.9°C, 29.0 ± 1.9°C, and 28.7 ± 1.9°C for the control, dynapenia, presarcopenia, and sarcopenia groups, respectively.

### Signal analysis

The baseline drift was eliminated by passing PPG signals through a digital 11th-order high-pass Chebyshev filter with a lower cutoff frequency of 0.01 Hz. To distinguish their beat-to-beat waveforms, two adjacent minimums of the signal were identified as the cut-off point for each pulse.

Each pulse (between two adjacent minimums) was represented by the following finite series (21, 22). The pulse was excluded if the value between the two adjacent minimums exceeded 20% of the average pulse amplitude.


$$\begin{array}{l} x\left(t\right)=\frac{A_{0}}{2}+\left\{\overset{k/2}{\underset{n=1}{\sum }}A_{n}\cos n\omega t_{s}+\overset{k/2}{\underset{n=1}{\sum }}B_{n}\cos n\omega t_{s}\right\} \end{array}$$

The Fourier coefficients (*A*_*n*_ and *B*_*n*_) of the pulse can be calculated as


$$\begin{array}{l} A_{n}=\frac{2}{k}\overset{k}{\underset{s=0}{\sum }}x_{s}\cos n\omega t_{s}\;\left(\text{for}\;n=0,1,...,\frac{k}{2}\right)\\ B_{n}=\frac{2}{k}\overset{k}{\underset{s=0}{\sum }}x_{s}\sin n\omega t_{s}\;\left(\text{for}\;n=0,1,...,\frac{k}{2}\right) \end{array}$$

where ω is the angular frequency and *t*_*s*_ is the sampling time interval.

The amplitude (*Amp*_*n*_) of each harmonic can then be calculated as $Amp_{n}=\sqrt{A_{n}^{2}+B_{n}^{2}}$. *Amp*_*n*_ is the amplitude of the nth harmonic of the PPG pulse harmonic spectrum.

Since the first 10 harmonics distributed most of the spectral energy of the pulse signal (23), the average value of PPG pulses was measured by pulse harmonic spectrum as the amplitude proportion (C_*n*_) of the *n*th harmonic component according to *Amp*_*n*_/*Amp*_0_ ×100% for *n* = 1–10. The standard deviation (SD_*n*_) and the coefficient of variation (CV_*n*_) of the nth harmonic component of amplitude proportion were also collected to evaluate the variability in the PPG beat-to-beat waveform.

In the pulse PPG waveform, lower-frequency components occupied larger proportions in the spectral power; therefore, the lower-frequency component can be more affected by and can hence help to reflect the changes in the transmission condition for the arterial pulse. In our previous unpublished study, we also found that lower-frequency components of the harmonic ratio can be more likely to be associated with the stiffness and elasticity of the vascular wall. Thus, we defined the vascular elasticity index (VEI) for each pulse as the ratio of patients to healthy individuals with harmonic ratio1 ^*^ harmonic ratio 2 of the PPG pulses during each 1-min measurement period.


$$\begin{array}{l} \frac{ratio1_{p}\times ratio2_{p}}{ratio1_{H}\times ratio2_{H}}=\frac{\text{ratio}1_{p}\times ratio2_{p}}{0.92\times 0.45} \end{array}$$

This study used MATLAB (MathWorks, Natick, MA, USA) for all signal processing and SPSS version 13.0 (SPSS Inc., Chicago, Illinois, USA) for all statistical analyses. The differences between basic physiological parameters and PPG parameters were calculated by one-way analysis of variance and Tukey's HSD method for *post-hoc* analysis. The independent influence of basic physiological parameters on PPG indicators was calculated by logistic regression analysis. The significance level was defined as *p* < 0.05.

## Results

A total of 170 elderly subjects (67 males and 103 females, age ≥60 years) met the inclusion criteria. A total of 102 of these individuals did not undergo PPG and were thus excluded. Sixty-eight of these individuals (29 males and 39 females, age 62–92 years) were enrolled in our study and finished the program. Nine of these individuals were diagnosed with sarcopenia according to the criteria of AWGS. The general characteristics and the other clinical or biochemical indicators of the participants are listed in Table 2. Subjects with sarcopenia had a lower BMI, lower abdominal circumference, and lower systolic pressure than those without sarcopenia, but the difference was not statistically significant. The prevalence of sarcopenia in women gender (8 out of 9, 88.9%, *p* = 0.046) and osteoporosis in dynapenia (7 out of 16, 43.8%, *p* = 0.005) were significant higher. Amplitude proportions and the variability indices of certain harmonic components within the PPG waveform are listed in Table 3, and they differed significantly or prominently between the sarcopenia and control groups. Among the four groups, the amplitude proportions were the largest in the sarcopenia group for harmonics 1, 2, 5, and 8. The differences between sarcopenia and non-sarcopenia groups were significant for harmonics 2, 5, 8, and 9. However, among the four groups, most of the SD_*n*_-values were larger in the sarcopenia group than in the other three groups. For instance, compared with control, dynapenia, and presarcopenia, sarcopenia had largest SD_*n*_-values for harmonics 1, 2, 3, and 5 (ratio 1, 2, 3, 5 = 0.354, 0.209, 0.137, 0.074); whereas sarcopenia had largest CV_*n*_-values for harmonics 1, 2, 3, and 10 (ratio 1, 2, 3, 10 = 0.263, 0.310, 0.402, 0.791).

**Table 2:** Characteristics of the study participants by control, dynapenia, presarcopenia, and sarcopenia.

**Characteristics of the study participants**	**Control**	**Dynapenia**	**Presarcopenia**	**Sarcopenia**	**Total**	***P*-value**
	***n* = 33**	***n* = 16**	***n* = 10**	***n* = 9**	***n* = 68**	
**Continuous variables**						
Age (years), mean (SD)	70.64 (4.993)	72.63 (6.612)	72.40 (4.993)	71.44 (3.468)	71.47 (5.222)	0.716
BMI (kg/m^2^), mean (SD)	24.636(2.746)	24.848 (2.222)	21.393 (2.157)	22.042(2.264)	23.866(2.798)	0.602
Abdominal circumference (cm), mean (SD)	81.397 (8.9673)	82.813 (7.6177)	72.500 (6.8191)	76.000(5.9582)	79.707 (8.6713)	0.380
Systolic blood pressure (mmHg), mean (SD)	137.27 (17.089)	132.19 (16.339)	132.10 (11.090)	125.89(13.986)	133.81(15.954)	0.149
Diastolic blood pressure (mmHg), mean (SD)	78.79 (10.816)	77.31 (11.768)	76.50 (7.605)	73.11 (12.574)	77.35 (10.820)	0.290
Sleep (h), mean (SD)	6.833 (1.157)	6.719 (1.183)	6.778 (0.972)	6.333 (0.866)	6.731 (1.095)	0.562
Pulse (/min), mean (SD)	72.85 (9.210)	73.94 (13.533)	81.40 (10.977)	72.44 (7.502)	74.31 (10.652)	0.209
**Continuous variables**						
Gender (male), *n* (%)	18 (54.5)	8 (50)	2 (20)	1 (11.1)	29 (42.6)	0.046
Hypertension, *n* (%)	11 (33.3)	9 (56.3)	3 (30)	3 (33.3)	26 (38.2)	0.306
Type 2 diabetes mellitus, *n* (%)	3 (9.1)	5 (31.3)	3 (30)	1 (11.1)	12 (17.6)	0.235
Malignancy, *n* (%)	4 (12.1)	1 (6.3)	2 (10)	3 (33.3)	10 (14.7)	0.276
Coronary heart disease, *n* (%)	2 (6.1)	2 (12.5)	1 (10)	1 (11.1)	6 (8.8)	0.644
Chronic obstructive pulmonary disease, *n* (%)	0 (0)	1 (6.3)	1 (10)	2 (22.2)	4 (5.9)	0.100
Smoking, *n* (%)	7 (21.2)	4 (25)	2 (20)	1 (11.1)	14 (20.6)	0.293
Osteoporosis, *n* (%)	3 (9.1)	7 (43.8)	3 (30)	0 (0)	13 (19.1)	0.005

**Table 3:** Characteristics of the study participants by control, dynapenia, presarcopenia, and sarcopenia.

**PPG indexes**	**Control**	**Dynapenia**	**Presarcopenia**	**Sarcopenia**	**Total**	***P*-value**
	***n* = 33**	***n* = 16**	***n* = 10**	***n* = 9**	***n* = 68**	
VEI (ratio 1 and 2), mean (SD/CV)	1.832(0.554/0.302)	1.788(0.720/0.403)	1.528(0.366/0.240)	2.332(1.479/0.634)	1.843(0.768/0.417)	0.007
Ratio1, mean (SD/CV)	1.232 (0.176/0.143)	1.230 (0.231/0.188)	1.147(0.143/0.125)	1.344(0.354/0.263)	1.234(0.217/0.176)	0.060
Ratio2, mean (SD/CV)	0.603(0.111/0.184)	0.583(0.138/0.237)	0.545(0.075/0.138)	0.673 (0.209/0.310)	0.599(0.132/0.220)	0.019
Ratio3, mean (SD/CV)	0.359(0.124/0.346)	0.307(0.105/0.341)	0.285(0.057/0.199)	0.341(0.137/0.402)	0.334(0.116/0.346)	0.362
Ratio4, mean (SD/CV)	0.189(0.063/0.334)	0.172(0.086/0.500)	0.151(0.042/0.277)	0.171(0.075/0.437)	0.177(0.068/0.385)	0.389
Ratio5, mean (SD/CV)	0.159 (0.046/0.286)	0.138 (0.064/0.460)	0.143(0.025/0.172)	0.165(0.074/0.447)	0.153(0.052/0.343)	0.021
Ratio6, mean (SD/CV)	0.118(0.045/0.377)	0.107(0.066/0.619)	0.086(0.027/0.315)	0.097(0.054/0.555)	0.108(0.050/0.464)	0.013
Ratio7, mean (SD/CV)	0.064(0.033/0.509)	0.068(0.051/0.751)	0.050(0.016/0.324)	0.052(0.029/0.560)	0.061(0.036/0.580)	0.090
Ratio8, mean (SD/CV)	0.041(0.015/0.359)	0.044(0.026/0.585)	0.040(0.009/0.212)	0.044(0.025/0.569)	0.042(0.018/0.437)	0.005
Ratio9, mean (SD/CV)	0.031(0.012/0.373)	0.037(0.027/0.733)	0.030(0.008/0.263)	0.032(0.023/0.719)	0.032 (0.017/0.538)	0.002
Ratio10, mean (SD/CV)	0.022 (0.011/0.500)	0.026 (0.019/0.712)	0.019 (0.009/0.475)	0.020(0.016/0.791)	0.023(0.014/0.604)	0.101

The results of the binary logistic regression analysis between groups by PPG indices are listed in Tables 4, 5. Following adjustment for potential confounders (age, sex, BMI), the Δ odds ratios of the ratio3, ratio4, and ratio6 tertile values were significantly increased in the sarcopenia and possible sarcopenia groups compared with the control group (*P* < 0.05) [ratio3 tertile: Δ odds = 0.499/0.469, *P* = 0.028/0.030; ratio4 tertile: Δ odds = 0.499/0.509, *p* = 0.028/0.049; ratio6 tertile: Δ odds = 0.499/0.514, *P* = 0.028/0.050; (unadjusted/adjusted)].

**Table 4:** Association between sarcopenia group by PPG indexes from logistic regression models.

**Unadjusted**	**Control (*****n*** = **33)**		**Nonsarcopenia** **(*****n*** = **59)**		**Adjusted**	**Control (*****n*** = **33)**		**Non-sarcopenia** **(*****n*** = **59)**	
	**vs**.		**vs**.			**vs**.		**vs**.	
	**Possible sarcopenia (*****n*** = **35)**		**Sarcopenia (*****n*** = **9)**			**Possible sarcopenia** **(*****n*** = **35)**		**Sarcopenia** **(*****n*** = **9)**	
	**Δ odds**	**P-value**	**Δ odds**	**P-value**		**Δ odds**	**P-value**	**Δ odds**	**P-value**
VEI (ratio 1 and 2)	1.038	0.906	2.069	0.080	VEI (ratio 1 and 2)	1.039	0.912	3.462	0.112
Ratio1	1.091	0.938	11.190	0.121	Ratio1	0.953	0.969	27.524	0.116
Ratio2	0.641	0.810	76.105	0.087	Ratio2	0.723	0.872	2785.396	0.062
Ratio3	0.016	0.090	1.883	0.832	Ratio3	0.021	0.154	82.474	0.234
Ratio4	0.005	0.176	0.207	0.782	Ratio4	0.021	0.371	56.010	0.526
Ratio5	0.007	0.302	127.494	0.456	Ratio5	0.007	0.314	944.272	0.312
Ratio6	0.000	0.108	0.005	0.498	Ratio6	0.001	0.212	0.195	0.853
Ratio7	0.009	0.505	0.000	0.392	Ratio7	0.134	0.791	0.001	0.650
Ratio8	63.956	0.756	355.466	0.757	Ratio8	16885.898	0.508	1493122.271	0.494
Ratio9	1443.577	0.610	0.093	0.911	Ratio9	23125967.867	0.296	2761.235	0.740
Ratio10	1.998	0.969	0.000	0.551	Ratio10	165974.921	0.547	0.000	0.809

**Table 5:** Association between sarcopenia group by PPG indexes from logistic regression models in tertile.

**Unadjusted**	**Control (*****n*** = **33)**		**Nonsarcopenia (*****n*** = **59)**		**Adjusted**	**Control (*****n*** = **33)**		**Non-sarcopenia** **(*****n*** = **59)**	
	**vs**.		**vs**.			**vs**.		**vs**.	
	**Possible sarcopenia** **(*****n*** = **35)**		**Sarcopenia (*****n*** = **9)**			**Possible sarcopenia** **(*****n*** = **35)**		**Sarcopenia** **(*****n*** = **9)**	
	**Δ odds**	**P-value**	**Δ odds**	**P-value**		**Δ odds**	**P-value**	**Δ odds**	**P-value**
VEI (ratio 1 and 2) (tertile)	1.665	0.354	3.937	0.039	VEI (ratio 1 and 2) (tertile)	1.853	0.320	6.956	0.030
Ratio1 (tertile)	0.729	0.296	1.446	0.415	Ratio1 (tertile)	0.676	0.250	2.041	0.181
Ratio2 (tertile)	0.665	0.181	1.184	0.703	Ratio2 (tertile)	0.699	0.290	1.893	0.233
Ratio3 (tertile)	0.499	0.028	0.802	0.619	Ratio3 (tertile)	0.469	0.030	1.075	0.887
Ratio4 (tertile)	0.499	0.028	0.657	0.354	Ratio4 (tertile)	0.509	0.049	0.791	0.644
Ratio5 (tertile)	0.799	0.454	0.975	0.954	Ratio5 (tertile)	0.845	0.607	1.258	0.660
Ratio6 (tertile)	0.499	0.028	0.657	0.354	Ratio6 (tertile)	0.514	0.050	0.789	0.638
Ratio7 (tertile)	0.550	0.056	0.419	0.082	Ratio7 (tertile)	0.557	0.085	0.444	0.128
Ratio8 (tertile)	0.955	0.878	0.802	0.619	Ratio8 (tertile)	1.097	0.779	0.856	0.749
Ratio9 (tertile)	0.799	0.454	0.802	0.619	Ratio9 (tertile)	0.879	0.696	0.875	0.774
Ratio10 (tertile)	0.729	0.296	0.657	0.354	Ratio10 (tertile)	0.838	0.592	0.786	0.632

The VEI of the PPG waveform in Table 3 shows that subjects with sarcopenia had significant higher VEI of the PPG waveform (mean = 2.332, SD = 1.479, CV = 0.634, *p* = 0.007) than the other three groups, and the results of the binary logistic regression analysis in Table 4 showed that VEI in the sarcopenia group appeared to be higher than in the non-sarcopenia groups (adjusted odds ratio, 2.069; *p* = 0.080, at the statistical borderline), whereas the results of the binary logistic regression analysis in tertile in Table 5 met statistical significance between the sarcopenia and non-sarcopenia groups whether adjusted or unadjusted (adjusted odds ratio 6.956, *p* = 0.030; unadjusted odds ratio 3.937, *p* = 0.039).

## Discussion

Sarcopenia, which is defined as the loss of skeletal muscle mass, strength and a decline of physical performance, is associated with an increased risk of cardiovascular disease (9–11). Because of the many identical risk factors between sarcopenia and cardiovascular disease, it could be deduced that there is an association between vascular dysfunction and muscle health. Current research only regards arterial stiffness as a manifestation of vascular dysfunction and its correlation with muscle tissue health (24).

In this study, it was found that certain harmonic components in amplitude proportions, harmonic variability, and VEI in the PPG waveform were significantly different between individuals with sarcopenia and healthy control subjects. The analysis of the four groups showed that there were no significant differences in the current PPG index between age groups. It could be assumed that the changes in these indices were partly attributed to hemodynamic changes caused by sarcopenia.

### VEI of the PPG waveform

Vascular elasticity index was defined according to the ratio of patients and healthy individuals based on harmonic ratio1 ^*^ harmonic ratio2 of the PPG pulses during each 1-min measurement period. In the pulse PPG waveform, lower-frequency components occupied larger proportions in the spectral power; therefore, the lower-frequency components can be more affected by and can hence help to reflect the changes in the transmission condition for the arterial pulse. Table 3 shows that subjects with sarcopenia had significantly higher VEI of the PPG waveform (mean = 2.332, SD = 1.479, CV = 0.634, *p* = 0.007) among the groups, and the results of the binary logistic regression analysis in Table 4 showed that VEI in the sarcopenia group was higher than those of the non-sarcopenia groups, but the difference did not meet statistical significance (adjusted odds ratio 2.069, *p* = 0.080). Meanwhile, the results of the binary logistic regression analysis in the tertiles in Table 5 met statistical significance between the sarcopenia and non-sarcopenia groups whether adjusted or unadjusted (adjusted odds ratio 6.956, *p* = 0.030; unadjusted odds ratio 3.937, *p* = 0.039). Sarcopenia is associated with alterations in the arterial system, such as changes in the vascular stiffness and resistance. It is possible that when faced with changes in vascular properties induced by sarcopenia, the cardiovascular system may need to perform more strongly in order to maintain the homeostasis of blood supply, and the sarcopenia subjects may thus have larger values of the harmonic variations. Therefore, VEI showed a significantly higher mean, SD and CV in the sarcopenia group, indicating poorer elasticity of the arteries, which could be partly inferred to be a sarcopenia-induced increase in arterial stiffness.

### Amplitude proportions of harmonic components (C*_*n*_*-values)

Table 3 shows that there were some significantly different amplitude proportions in certain harmonic components between the sarcopenia group and other groups. Among the four groups, the largest harmonic components in sarcopenia group were harmonics 1, 2, 5, and 8 and the differences between sarcopenia and non-sarcopenia groups were significant in harmonics 2, 5, 8, and 9.

The arterial blood pressure pulse is produced by the driving force of the heartbeat and is transmitted to the peripheral vascular bed through the arteries. The texture along the blood vessel wall may affect pulse formation during transmission, thereby affecting the pulse shape (25–27). A recent study focused on developing indices via pulse analysis to distinguish subjects with different diseases which may induce changes along blood vessels. For example, diseases including hypertension, diabetes, stroke, and inflammatory rheumatic diseases have been studied to detect the effects of diseases on arterial elasticity or peripheral vascular resistance by BP pulse velocity and various waveform index analyses (28–30).

Due to the quasiperiodicity of the heartbeat, harmonic analysis has become an ideal method in the study of cardiovascular signals, including blood pressure and PPG waveforms, in many frequency domain analysis methods (17, 18). For instance, previous research has suggested that harmonic amplitude analysis has potential application value in detecting changes in blood vessel elasticity (20, 31). Studies have shown that each vascular bed corresponds to an independent, specific frequency, and linear cumulative effect of the peripheral pressure wave. The harmonic components of arterial BPW are associated with changes in the elasticity of the local vascular bed (31). Since different diseases could cause damage to different local vascular beds (such as changes in diameter or elasticity) that reflect changes in the hydrodynamics of vascular flow detected by harmonic components of arterial BPW, the amplitude distribution of harmonic components may vary from disease to disease. Our previous study reported that changes in the harmonic distribution could be applied to detect arterial elasticity in stroke patients (21).

### Variability of harmonics in pulse contour spectrum (SD*_*n*_*- and CV*_*n*_*-values)

The variability of some harmonic components in the PPG waveform was significantly different between the sarcopenia group and the control groups, as shown in Table 3. Among the four groups, most of the SD_*n*_-values were larger in the sarcopenia group than in the other three groups. For example, compared with control, dynapenia, and presarcopenia, sarcopenia had significantly larger SD_*n*_-values for harmonics 1, 2, 3, and 5 and relatively larger SD_*n*_-values for harmonics 4, 6, 8, 9, and 10, whereas sarcopenia had significantly larger CV_*n*_-values for harmonics 1, 2, 3, and 10 and relatively larger CV_*n*_-values for harmonics 4, 5, 6, 7, 8, and 9.

Several underlying mechanisms of BP variability with aging have been proposed, such as hemodynamic instability, advanced artery remodeling and atherosclerosis, arterial stiffness, baroreflex impairment, endothelial dysfunction, and subclinical inflammation (32). A general loss of cardiovascular compliance contributes to reduced homeostatic adaptation to stress, as evidenced by altered physiologic dynamics of multiple physiological processes, notably the stability of the cardiovascular and nervous systems or the pulsatile release of hormones (33). Blood pressure variability might help predicting the onset of several age-related conditions and diseases, independently of chronological age. A recent study by Uçar et al. investigated a low-cost and trustworthy Body Muscle Percentage calculation model based on artificial intelligence algorithms and biomedical signals demonstrating that PPG-based models can be used to predict body muscle percentage and consequently sarcopenia (34). Furthermore, studies of the variability of harmonics in the BPW spectrum (BPHV) have analyzed the effects of diseases (such as stroke, polycystic ovary syndrome, and metabolic syndrome) on the cardiovascular system in patients compared with healthy subjects (21, 35, 36).

Table 3 reveals that several harmonic components varied significantly between the sarcopenia group and other groups. As we assumed in the previous study, lower-frequency components of the harmonic ratio can be more likely to be associated with stiffness and elasticity of the vascular wall, and VEI was significantly larger in the sarcopenia group compared with the other groups. A recent study by Hashimoto et al., investigated 146 patients aged ≥65 years with diabetes from the KAMOGAWA-DM cohort study, without physical inactivity demonstrating that CV of systolic BP, rather than average systolic BP, is associated with sarcopenia in older patients with diabetes, which indicated that sarcopenia may induce arterial stiffness and thus increased arterial resistance (37). Due to the changes in blood vessels caused by sarcopenia, compensation of cardiovascular system may be activated to maintain proper blood supply, and thus reflecting larger values in lower-frequency components of harmonic ratio. Therefore, the current research results support the suggestion that the harmonic component index variability of the PPG waveform could be a useful tool to detect systematic changes in vascular characteristics caused by sarcopenia.

The results of the current study revealed significant differences between the groups, and in these PPG indices, there was little increase or decrease between the control group and the sarcopenia group. Compared with the sarcopenia stage, the arterial pulse transmission conditions changed less in the presarcopenia stage (this may be related to the less noticeable change in C_*n*_). In presarcopenia, due to the relatively healthy condition, the cardiovascular system may have more space for compensatory regulation, reducing the changes in vascular properties. The cardiovascular system adapts more easily, so in presarcopenia, the CV_*n*_ of many harmonic components is smaller than that in sarcopenia.

There are some limitations in this study. (1) The number of included subjects was relatively small. (2) There was some selection bias, as we included individuals who had voluntary geriatric health exams, not individuals from the general population. (3) There were different distributions of other confounding factors in each group that may have influenced our results, such as differences in sex, age, BMI, and the use of chronic disease medications. (4) A retrospective, cross-sectional study design limits the strength of evidence.

Tables 4, 5 show that most of the correlations between PPG indicators and basic physiological parameters were not significant, indicating that various basic physiological parameters (except sex and osteoporosis status) had little interference with PPG indicators. Further investigations are needed to verify how PPG harmonic parameters are affected by sarcopenia to understand the underlying mechanisms.

## Conclusion

The lower-frequency components of the harmonic ratio in which we defined VEI showed the significantly highest mean, SD, and CV in patients with sarcopenia, indicating poorer elasticity of the arteries that could be partly inferred to be a sarcopenia-induced increase in arterial stiffness. The present findings showed that finger PPG waveform measurements may be useful for the early detection of vascular diseases in patients with sarcopenia, and this non-invasive and easy-to-perform technique may expand in clinical applicability in the future.

## Data availability statement

The original contributions presented in the study are included in the article/supplementary material, further inquiries can be directed to the corresponding author/s.

## Ethics statement

The studies involving human participants were reviewed and approved by TSGHIRB 2-108-05-161. The patients/participants provided their written informed consent to participate in this study.

## Author contributions

TOY performed the analysis and wrote the paper. L-WW and HH conceived and designed the analysis. T-CP and W-LC contributed data or analysis tools. All authors contributed to the article and approved the submitted version.

## Funding

This work was supported by a grant to L-WW (TSGH-A-111013, TSGH-A-110005, and MND-MAB-110-129).

## Conflict of interest

The authors declare that the research was conducted in the absence of any commercial or financial relationships that could be construed as a potential conflict of interest.

## Publisher's note

All claims expressed in this article are solely those of the authors and do not necessarily represent those of their affiliated organizations, or those of the publisher, the editors and the reviewers. Any product that may be evaluated in this article, or claim that may be made by its manufacturer, is not guaranteed or endorsed by the publisher.
